# 445. An Ongoing Battle from a Forgotten War: Long COVID in Mississippi, October 2021 - September 2022

**DOI:** 10.1093/ofid/ofad500.515

**Published:** 2023-11-27

**Authors:** Manuela Staneva, Thomas Dobbs

**Affiliations:** Mississippi State Department of Health, Hattiesburg, Mississippi; University of Mississippi Medical Center, Jackson, Mississippi

## Abstract

**Background:**

Due to the lack of a well-established definition, long COVID—unlike its acute counterpart—has been evading statewide surveillance systems. In October 2021, however, the adoption of an ICD-10-CM code for long COVID, created the prerequisite needed for such retrospective state-level surveillance of long COVID. To seize this opportunity and establish baseline information, we used Mississippi’s hospital discharge data to examine hospitalizations associated with long COVID.

**Methods:**

Hospital discharge data are a population-level data source that captures data from all non-federal hospitals in the state and contains information on diagnoses, procedures, and resource utilization. In this cross-sectional study, we utilized the latest available data that incorporates long COVID diagnoses (10/01/2021-09/30/2022). The study included only adult Mississippi residents (≥ 18 years) and the unit of analysis was individual patients. We performed descriptive and inferential (chi-squared tests) statistical analyses.

**Results:**

Among the 1,213 patients hospitalized with post-COVID-19 complications, the majority were white (69.7%), elderly (average age = 66), rural residents (60.3%), and females (53.8%). The most common comorbidities were hypertension (73.6%), diabetes (39.4%), chronic pulmonary disease (28.4%), and congestive heart failure (28.4%). Among long haulers, 22.1% were admitted to ICU, 13.6% needed respiratory support, and 8.5% died in the hospital. The in-hospital mortality proportion among long haulers did not differ by race, residence, or insurance status. In-hospital deaths were more likely to occur, however, among patients ≥ 65 years (68.7% vs. 56.3%, p < .01). Compared to survivors, deceased patients with long COVID had a slightly higher average number of comorbid conditions (2.8 vs. 2.2). For patients with long COVID, the mean length of stay was 11 days and the mean hospital charges were $109,739.

**Conclusion:**

In our study, we identified that hypertension was highly prevalent among long haulers. The severity of illness as measured by ICU care, in-hospital mortality, length of stay, and hospital charges was also high among patients with long COVID. These findings underscore the importance of long-term surveillance of COVID-19 complications.

**Disclosures:**

**All Authors**: No reported disclosures

